# Ticks and Tick-Borne Pathogens Circulating in Peri-Domestic Areas in Mainland Portugal

**DOI:** 10.3390/microorganisms12051006

**Published:** 2024-05-16

**Authors:** Leonardo Moerbeck, Ricardo Parreira, Magdalena Szczotko, Gonçalo Seixas, Rita Velez, Małgorzata Dmitryjuk, Ana Sofia Santos, Ana Domingos, Sandra Antunes

**Affiliations:** 1 Instituto de Higiene e Medicina Tropical (IHMT), Universidade NOVA de Lisboa (UNL), Rua da Junqueira 100, 1349-008 Lisboa, Portugal; ricardo@ihmt.unl.pt (R.P.); gseixas@ihmt.unl.pt (G.S.); adomingos@ihmt.unl.pt (A.D.); 2 Global Health and Tropical Medicine (GHTM), Associate Laboratory in Translation and Innovation Towards Global Health (LA-REAL), Instituto de Higiene e Medicina Tropical (IHMT), Universidade NOVA de Lisboa (UNL), Rua da Junqueira 100, 1349-008 Lisboa, Portugal; 3 Students’ Parasitology “Vermis” Science Club, Department of Medical Biology, Collegium Medicum, School of Public Health, University of Warmia and Mazury in Olsztyn, 10-719 Olsztyn, Poland; magdalena.szczotko@uwm.edu.pl; 4 Department of Biochemistry, Faculty of Biology and Biotechnology, University of Warmia and Mazury in Olsztyn, Oczapowskiego 1A, 10-719 Olsztyn, Poland; m.dmit@uwm.edu.pl; 5 Centro de Estudos de Vetores e Doenças Infeciosas Dr. Francisco Cambournac, Instituto Nacional de Saúde Doutor Ricardo Jorge (CEVDI-INSA), 2965-575 Águas de Moura, Portugal; rita.perdigao.velez@gmail.com (R.V.); ana.santos@insa.min-saude.pt (A.S.S.); 6 Instituto de Saúde Ambiental, Faculdade de Medicina, Universidade de Lisboa, 1649-004 Lisboa, Portugal

**Keywords:** tick-borne pathogens, *Ixodes ricinus*, *Neoehrlichia mikurensis*, surveillance, One Health

## Abstract

Over the years, tick-borne pathogens (TBPs) have garnered significant interest due to their medical, veterinary and economic importance. Additionally, TBPs have drawn attention to how these microorganisms interact with their own vectors, increasing the risk to human and animal infection of emerging and reemerging zoonoses. In this sense, ticks, which are obligate hematophagous ectoparasites, have a key role in maintaining and transmitting TBPs among humans and animals. The aim of this study was to assess the prevalence of neglected TBPs in mainland Portugal, namely *Anaplasma* spp., *Babesia* spp., *Ehrlichia* spp. and *Neoehrlichia mikurensis*. DNA fragments were detected in questing ticks collected from five different ecological areas under investigation. To the best of the authors’ knowledge, this study reports new worldwide findings, including *B. bigemina* infecting *Ixodes frontalis*, *Ixodes ricinus* and *Rhipicephalus sanguineus* sensu lato. Additionally, it presents new findings in Portugal of *N. mikurensis* infecting *I. ricinus* and of presumably *Wolbachia* endosymbionts being detected in *I. ricinus*. Overall, there were 208 tick samples that were negative for all screened TBPs. The results herein obtained raise concerns about the circulation of neglected TBPs in mainland Portugal, especially in anthropophilic ticks, highlighting the importance of adopting a One Health perspective.

## 1. Introduction

Tick-borne diseases (TBDs) are emerging infections caused by a large spectrum of tick-borne pathogens (TBPs), encompassing bacteria, protozoa and viruses, which have become a global public health concern, especially because some of these pathogens cause zoonoses [1]. As these pathogens depend not only on vertebrate hosts but also on arthropod vectors, the prevalence of TBDs is closely linked to the distribution of the latter [2]. Therefore, the identification of the vector and its abundance, as well as the knowledge regarding associated pathogens, are key to assessing transmission risks and understanding disease transmission dynamics [3]. Ticks are obligate hematophagous ectoparasites that belong to the sub-order Ixodida, which is formed by two major tick families: Ixodidae, or “hard ticks”, comprising ticks with a dorsal scutum or shield, and Argasidae, or “soft ticks”, which lack this dorsal structure [4]. To date, there are approximately 900 described species from both major families, and 22 hard tick species have been reported in Portugal [5].

Since 2011, a nationwide vector surveillance network called REVIVE has been monitoring, among other arthropods, the activity of ticks and the most frequent TBP, namely *Rickettsia* spp. and *Borrelia* spp., widely describing infecting ticks in Portugal (https://www.insa.min-saude.pt/category/areas-de-atuacao/doencas-infeciosas/revive-rede-de-vigilancia-de-vetores/, accessed on 8 April 2024). However, other TBPs that have an impact on human and animal health, such as *Anaplasma phagocytophilum*, *Ehrlichia* spp., *Neoehrlichia mikurensis*, *Babesia* spp. and *Coxiella burnetii*, are not monitored by this surveillance network.

The agent of human granulocytic anaplasmosis (HGA), *A. phagocytophilum*, is a widely distributed zoonotic bacterium that is transmitted by different tick species [6,7]. In Portugal, *I. ricinus* has been identified as its vector, and it has also been reported to infect *Ixodes ventalloi* [5]. This agent causes a febrile syndrome characterized by a headache, anorexia, malaise and myalgias. Although HGA is usually regarded as a self-limited disease, it may become a severe illness and progress to human deaths, especially in individuals with comorbid conditions [8]. In addition, occasional infections in humans with *Anaplasma platys*, typically linked with dogs and *Rhipicephalus sanguineus* sensu lato, were recently reported in the USA [9].

The etiological agent of human monocytic ehrlichiosis (HME), *Ehrlichia chaffeensis*, causes an infection with a severe flu-like febrile syndrome, frequently showing signs similar to those of hepatitis, in patients within the USA [10]. In 1991, Portugal had its first and thus far only reported case of HME based on serological evidence [11]. Besides humans, dogs are also affected by *E. chaffeensis* and other *Ehrlichia* species. In 1992, the agent of canine granulocytic ehrlichiosis (CGE), *E. ewingii*, was first described, causing a new canine disease [12]. Seven years later, in the USA, it was reported to cause fevers, headaches and thrombocytopenia with or without leukopenia in four human patients [13], and it is presently the second cause of ehrlichiosis in this country [14]. *E. canis*, traditionally associated with a moderate-to-severe disease in dogs, has also been implicated in rare cases of human infection [15]. In addition, new human pathogens continue to arise from this taxon, such as *Ehrlichia muris eauclarensis* and *Ehrlichia ruminatum*, closely related to Panola Mountain *Ehrlichia* [16,17]. In the USA, these bacteria are mainly transmitted by ticks such as *Amblyomma americanum* (*E. chaffeensis*, *E. ewingii* and Panola Mountain *Ehrlichia*), *R. sanguineus* s. l. (*E. canis*) and *Ixodes scapularis* (*E. muris*) [16,17].

The bacterium *Neoehrlichia mikurensis* was first described infecting *Ixodes ovatus* and *Rattus norvegicus* in Japan [18]. Later, in 2009, it became a recognized zoonotic pathogen, when a 77-year-old patient was diagnosed presenting febrile episodes, an erysipela-like rash and thromboembolic complications [19]. Most recently, in 2019, this TBP was successfully cultivated and isolated [20]. The main vector of *N. mikurensis* in Europe is *I. ricinus* [21], and in Portugal, a *Neoehrlichia mikurensis*-like organism was found infecting *I. ventalloi* [22].

Human babesiosis is caused by intraerythrocytic protozoan parasites of the genus *Babesia*. Most reported cases of human babesiosis in Europe are caused by *Babesia divergens*, with fewer cases attributed to *Babesia microti* and *Babesia venatorum* [23]. Infections caused by *B. divergens* are characterized by septic fevers, severe anemia, hemoglobinuria and jaundice due to widespread hemolysis [23]. When comparing zoonotic infections among *Babesia* spp., those caused by *B. microti* and *B. venatorum* seem to be less aggressive. In addition, the primary vector of these *Babesia* spp. in Europe is *I. ricinus* [23].

*Coxiella burnetii* is the etiological agent of Q fever. This zoonotic bacterium is an obligate intracellular, Gram-negative, γ-proteobacteria, with a worldwide distribution [24]. First reported in Portugal in 1948 [24], the prevalence of anti-*C. burnetii* antibodies and the circulation of different genotypes of *C. burnetii* in domestic ruminants and wild animals have been investigated by several studies [25,26,27]. Furthermore, ticks were also found to be infected, showing that Ixodidae may play a role in the maintenance or in the transmission of this bacterium [22].

The epidemiology of TBPs and the distribution of ticks are intertwined, directly influenced by several factors, such as anthropophilic behavior, demographics, climate changes and wildlife population. Many TBPs are maintained in sylvatic cycles [28], and wild animals may act as reservoirs, amplifiers or even sentinel hosts for human infections [29]. The spill-over of these cycles into peri-domestic areas and the potential exposure of domestic animals and humans are particularly important. In this context, ticks with more permissive feeding behavior, such as *I. ricinus*, play a key role [3]. In fact, *I. ricinus* is not only one of the most significant tick species with vector competence and with the potential to act as reservoirs for TBPs in Europe [30], but it is also one of the primary species encountered on humans in Portugal [31]. Bearing that in mind, the objective of this study was to screen TBPs in peri-domestic and recreational areas at the interface between wild and domestic animals and humans, where *I. ricinus* occurs. A convenience sampling approach ensured that other tick species collected in these areas were also considered. The study was focused on TBPs that were still not actively surveilled in Portugal, such as *Anaplasma* spp., *Ehrlichia* spp., *Babesia* spp., *N. mikurensis* and *C. burnetti*, despite their potential relevance to both human and animal health.

## 2. Materials and Methods

**Figure 1:**
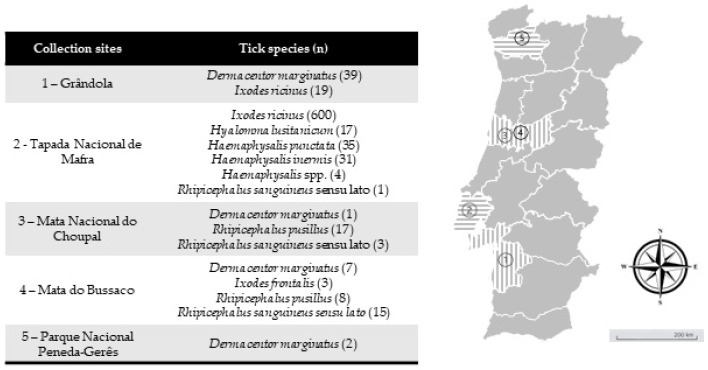
Collection sites of questing ticks collected in mainland Portugal. (n) = total number of tick specimens collected in a correspondent area. Map image adapted from Google Earth v.7.3.2.5491.

*Study site and collection of ticks*—Between February 2019 and May 2021, questing ticks were collected in mainland Portugal, comprising five different ecological areas with a known background of *I. ricinus* circulation. Tick collection occurred in different months at different locations. The first was Grândola (38°06′19.6″ N 8°33′59.7″ W) in February 2019, an area populated by cork oak and holm oak constituting a Montado habitat, which is a heterogeneous habitat created by man by adaptation of the Mediterranean forest [32]. Located in southern Portugal, it presents a Mediterranean climate characterized by hot, dry summers and mild, wet winters. The second was Mata Nacional do Choupal (40.2223° N, 8.4439° W) in June 2019. Located in central-northern Portugal near Coimbra city, it also presents a Mediterranean climate characterized by a mixed woodland of mostly deciduous broadleaf trees [33]. The third was Parque Nacional da Peneda-Gerês (41.7282° N, 8.1626° W) in December 2019. The park is in the north of Portugal and falls within a transitional zone between the Atlantic and Mediterranean environments, characterized by cold and rainy winters and warm summers. The area supports a diversity of habitats, including agricultural, shrublands and oak forest patches [34]. The fourth was Mata do Bussaco (40.3771° N, 8.3669° W) in June 2019. This forest is in central Portugal. With a climate that is predominantly Mediterranean but that presents some Atlantic influence, it constitutes an old-growth mixed woodland [35]. Finally, Tapada Nacional de Mafra (38.9646° N, 9.3027° W) was visited in December 2019 and May 2021 (Figure 1). This last site is located in the district of Lisbon on the west coast of Portugal. A Mediterranean climate characterizes this area. It encompasses various habitats, including woodlands, meadows, wetlands and streams, which support diverse flora and fauna [36]. All ecological areas were visited once, whereas at Tapada Nacional de Mafra, two collection efforts were made. All areas were screened on behalf of project PTDC/SAU-PAR/28947/2017. Ticks were collected by flagging and dragging vegetation. Up to 20 tick specimens were placed in a single 15.0 mL tube with some green vegetation to avoid tick dehydration, and they were kept refrigerated until laboratory arrival and processing. Ticks were taxonomically classified to the species level based on morphological characteristics according to previously published taxonomic keys and descriptions related to hard ticks in Europe and North Africa [37] using a Motic SMZ171 stereomicroscope (Kowloon, Hong Kong, China).

*Nucleic acid isolation*—After identification, specimens were rinsed in sterile phosphate-buffered saline solution (PBS) with a pH of 7.0 and were separated according to species, development stage, sex, date and place of collection. Subsequently, ticks were either processed individually when at the adult stage (female or male) or in pools of, at most, five nymphs in a sterile 1.5 mL tube. Ticks were frozen in liquid nitrogen and crushed with sterile mortars and pestles. Both DNA and RNA were extracted using TRIzol^TM^ Reagent (ThermoFisher Scientific, Carlsbad, CA, USA), according to the manufacturer’s protocol for the isolation of nucleic acids from tissues. Both nucleic acids were resuspended in nuclease-free water. The concentration of each sample was fluorometrically evaluated using a Qubit4 fluorometer (ThermoFisher Scientific, Carlsbad, CA, USA). To assess the absence of PCR inhibitors, 20% of all samples were randomly selected for amplification of the tick 18S *rDNA* gene fragment [38].

*PCR screening of TBP*—Conventional PCR assays used 5.0 μL of DNA samples with Piro-A (forward) and Piro-B (reverse) primers to amplify a 408 bp fragment of the small subunit of the 18S *rDNA* gene of the Order Piroplasmida, including *Babesia* spp. and *Theileria* spp. [39]. For *Anaplasma* spp. and *Ehrlichia* spp. detection, EHR16SD (forward) and EHR16SR (reverse) primers were used to amplify a 345 bp fragment of the 16S *rRNA* gene of bacteria belonging to the family Anaplasmataceae [40]. PCR assays were performed in 25.0 μL reactions with Supreme NZYTaq 2× Green Master Mix (NZYTech, Lisbon, Portugal) and 1.0 μM of each primer in a T100 thermal cycler (Bio-Rad, Hercules, CA, USA). To detect *C. burnetti* DNA, TaqMan real-time qPCR was performed using Cox-F (forward), Cox-R (reverse) primers and a probe (Cox-TM) to amplify a 295bp fragment of the repetitive insertion element *IS1111a* [41]. Reactions of 10.0 µL were performed in triplicate using NZYSupreme qPCR Probe Master Mix in a CFX Connect Real-Time PCR Detection System (Bio-Rad, Hercules, CA, USA). To detect *N. mikurensis*, total extracted RNA was used for cDNA synthesis using the iScript cDNA Synthesis Kit (Bio-Rad, Hercules, CA, USA) according to the user´s guide protocol. qPCR was performed using NEO_16S_F (forward) and NEO_16S_R (reverse) primers to amplify a 107 bp fragment of the 16S *rRNA* gene [42]. All qPCR reactions to detect *N. mikurensis* were prepared in 10.0 µL triplicates on a 96-well plate using iTaq Universal SYBR Green Supermix (Bio-Rad, Hercules, CA, USA) in a CFX Connect Real-Time PCR Detection System (Bio-Rad, Hercules, CA, USA). Samples that were considered positive in qPCR screening assays were those for which at least two or more of their replicates yielded a positive amplification. All primers used for pathogen detection are listed in Table 1. PCR positive controls included DNA extracted from an in-house *Babesia ovis* (Israeli strain) culture, *A. phagocytophilum* Webster strain (Focus Diagnostics, Cyperss, CA, USA), *C. burnetii* Nine Mile strain (Vircell Microbiologists, Granada, Spain) and a synthetized gBlocks^®^ Gene Fragment (IDT-Integrated DNA Technologies, Leuven, Belgium) encompassing a 106 bp *N. mikurensis* 16S ribosomal gene.

*DNA Sequencing*—Amplicons from standard PCR were purified using the NZYGelpure kit (NZYtech, Lisbon, Portugal) according to the user’s guide protocol, and they were sent to StabVida (Caparica, Portugal), where Sanger sequencing was performed. All obtained sequences were aligned, compared to those deposited at the NCBI (National Center for Biotechnology Information) nucleotide database (https://blast.ncbi.nlm.nih.gov/). Obtained sequences corresponding to the 18S *rDNA* amplicon were deposited at GenBank under accession numbers PP346439-PP346444, and those corresponding to 16S *rRNA* were assigned accession numbers PP346424-PP346433.

*Phylogenetic analysis*—All datasets were created with reads obtained as a result of Sanger sequencing; reference sequences previously deposited in GenBank; and sequences returned from Megablast search [Nucleotide BLAST: Search nucleotide databases using a nucleotide query (nih.gov)] that demonstrated the best “query cover” and “identity percentage” rates, always from different studies. All sequences in each gene-specific given dataset were aligned using MAFFT (https://mafft.cbrc.jp/alignment/server/), with known sequences previously deposited in GenBank. Multiple alignments were edited on the online server GBlocks 0.91b (http://phylogeny.lirmm.fr/phylo_cgi/one_task.cgi?task_type=gblocks). Two different approaches were used to construct the phylogenetic trees to minimize the bias of the results by the selected method. At first, phylogenetic trees were constructed based on neighbor-joining (NJ) analysis with the Kimura two-parameter (K2P) substitution model with MEGA v.10 [43]. Afterward, another set of phylogenetic trees was constructed based on maximum likelihood (ML) analysis using the best-fit model for each sequence dataset, according to the BIC (Bayesian Information Criterion), as defined by IQ-TREE web server model selection (http://iqtree.cibiv.univie.ac.at/). The topographic representation that displayed the phylogenetic trees selected for the current study was that from the NJ analysis once the outcomes of the phylogenetic analyses using both approaches (NJ and ML) demonstrated identical topological characteristics, i.e., the same nodes, branches and bootstrap values that were obtained from 1000 randomly generated trees.

## 3. Results

A total of 802 questing ticks belonging to eight species were obtained: *Dermacentor marginatus* (n = 49), *Haemaphysalis inermis* (n = 31), *Haemaphysalis punctata* (n = 35), *Hyalomma lusitanicum* (n = 17), *Ixodes frontalis* (n = 3), *I. ricinus* (n = 619), *R. pusillus* (n = 25) and *Rhipicephalus sanguineus* sensu lato (n = 19) (Table 2). In addition, four *Haemaphysalis* spp. remained with no species identification.

For the screening of TBPs, ticks were processed in a total of 365 samples. The obtained prevalence for piroplasmids was 18.63% (n = 68), and for Anaplasmataceae microorganisms, it was 29.04% (n = 106). For *N. mikurensis*, 178 samples were screened, corresponding to a prevalence of 14.04% (n = 25). A total of 11 samples that were positive for *N. mikurensis* corresponded to *I. ricinus*, 4 samples corresponded to *D. marginatus*, 3 samples corresponded to *R. pusillus*, and 7 samples corresponded to *R. sanguineus* s. l. ticks. All samples screened for *C. burnetii* were negative. It is important to note that some positive samples (n = 63 for piroplasmids and n = 96 for Anaplasmataceae bacteria) were deemed positive in the context of tick infection, although with no phylogenetic resolution (Table 2).

**Figure 2:**
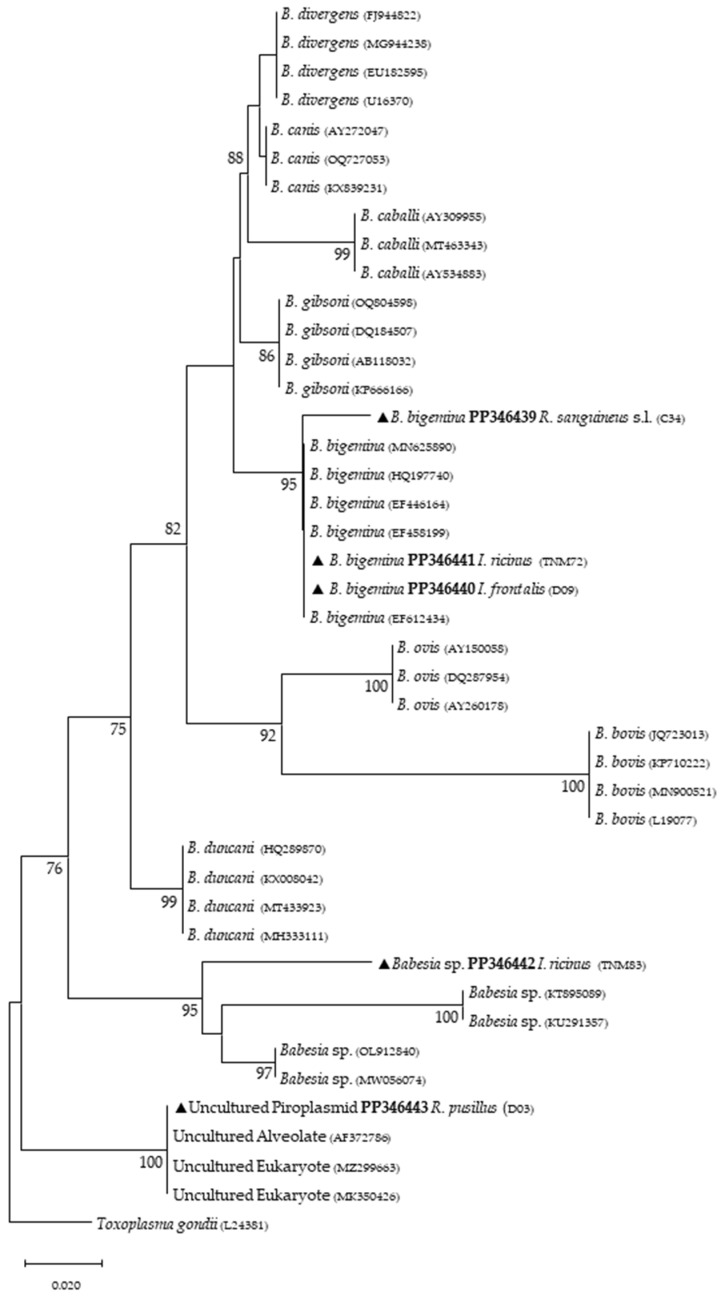
Phylogenetic tree constructed and displayed by the neighbor-joining method and Kimura’s two-parameter evolution model from partial sequences of the 18S *rRNA* gene. The same topographic representation was obtained by the maximum likelihood method and Kimura’s two-parameter + G4 evolution model from partial sequences of the 18S *rRNA* gene according to the BIC (Bayesian Information Criterion), as defined by IQ-TREE web server model selection. Bootstrap values were obtained from 1000 replications and are indicated at the nodes of the respective branches (only values ≥ 75%). All piroplasmid sequences obtained during this work are highlighted with a triangle and its respective accession, both in bold format.

**Figure 3:**
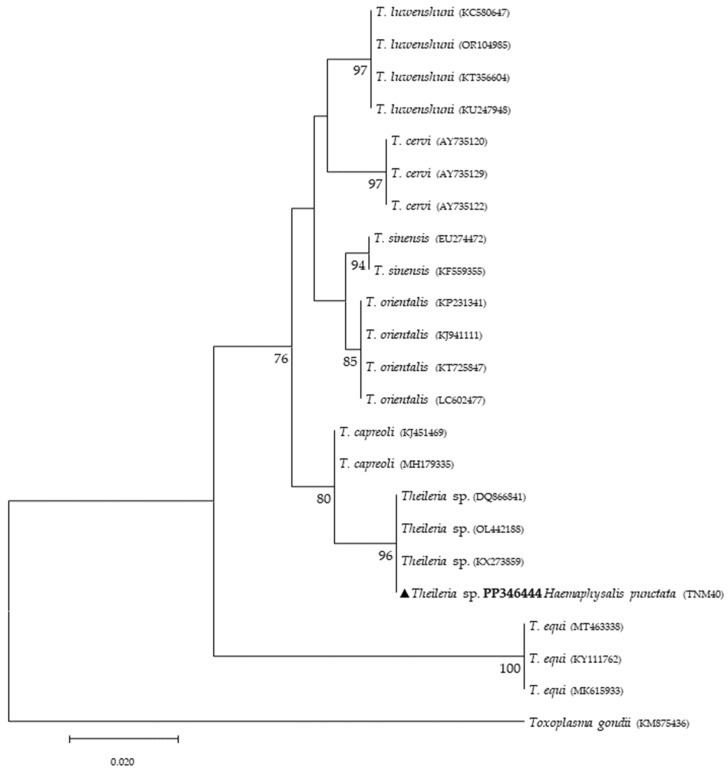
Phylogenetic tree constructed and displayed by the neighbor-joining method and Kimura’s two-parameter evolution model from partial sequences of the 18S *rRNA* gene. The same topographic representation was obtained by the maximum likelihood method and Kimura’s two-parameter + I + G4 evolution model from partial sequences of the 18S *rRNA* gene according to the BIC (Bayesian Information Criterion), as defined by IQ-TREE web server model selection. Bootstrap values were obtained from 1000 replications and are indicated at the nodes of the respective branches (only values ≥ 75%). The *Theileria* sp. sequence obtained during this work is highlighted with a triangle and its respective accession, both in bold format.

**Figure 4:**
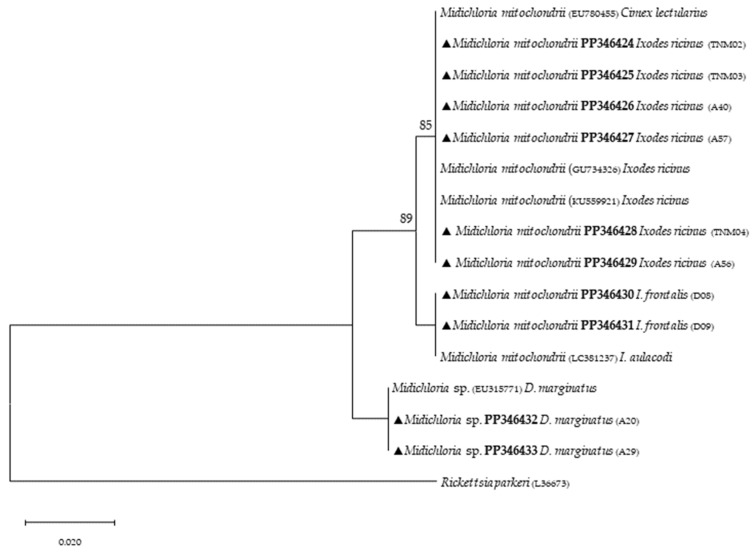
Phylogenetic tree constructed and displayed by the neighbor-joining method and Kimura’s two-parameter evolution model from partial sequences of the 16S *rRNA* gene. The same topographic representation was obtained by the maximum likelihood method and Kimura’s two-parameter evolution model from partial sequences of the 16S *rRNA* gene according to the BIC (Bayesian Information Criterion), as defined by IQ-TREE web server model selection. Bootstrap values were obtained from 1000 replications and are indicated at the nodes of the respective branches (only values ≥ 75%). All *Midichloria* spp. sequences obtained during this work are highlighted with a triangle and its respective accession, both in bold format.

Phylogenetic analysis based on 18S *rRNA* gene fragments for piroplasmids showed *B. bigemina* infecting three different tick species: *I. frontalis* (a single nymph) (PP346440), *I. ricinus* (pool of five nymphs) (PP346441) and *R. sanguineus* s. l. (one adult male) (PP346439). *Babesia* sp. was found infecting an *I. ricinus* sample (pool of five nymphs) (PP346442), and one unknown piroplasmid was found infecting a *R. pusillus* sample (one female) (PP346443) (Figure 2). It is important to mention that the result obtained from the Megablast analysis of this sample, unlike the others, did not return a single identity but rather several possible ones, none of which were clear. Additionally, *Theileria* sp. was infecting one female *H. punctata* (PP346444) tick (Figure 3). Phylogenetic analysis based on 16S *rRNA* gene sequences amplified using Anaplasmatacea bacteria-specific primers revealed the presence of *Midichloria mitochondrii* DNA in ten samples, with two corresponding to *D. marginatus* (PP346432-33), two corresponding to *I. frontalis* (PP346430-31) and six corresponding to *I. ricinus* (PP346424-29) (Figure 4). Furthermore, due to the length constraints of the obtained sequences from *I. ricinus* ticks, the existence of *Wolbachia* endosymbionts was hypothesized but unable to be confirmed.

Among positive samples, co-infections were detected in ten samples (adult ticks). Two *I. ricinus* from Grândola, two from TNM, one *D. marginatus* from Bussaco and one *R. pusillus* from Mata do Choupal were found positive for both *N. mikurensis* and piroplasmids. Three *I. ricinus* from TNM, two *D. marginatus* from Bussaco and one *R. pusillus* from Mata do Choupal were found positive for *N. mikurensis* and Anaplasmataceae. However, since it was not possible to identify piroplasmids and other Anaplasmataceae species, these associations need to be further evaluated.

## 4. Discussion

Eight different tick species were collected from the vegetation in the collection sites. All tick species have been previously reported in Portugal [5]. *I. ricinus* was not only the most prevalent tick species but also the most frequently collected among immature stages. In accordance with previous studies [5], *I. ricinus* can be considered a common tick since it has been recorded in eleven out of the eighteen Portuguese administrative regions (districts). It is important to highlight that the TNM area was the main contributor of this result. The high prevalence of this generalist tick in a natural reserve park, together with the circulation of human TBPs, pose a relevant health risk.

Regarding tick infections, *B. bigemina* was the most frequent piroplasmid detected in different tick species, namely *I. frontalis*, *I. ricinus* and *R. sanguineus* s. l. As observed in Figure 2, the obtained *B. bigemina* 18S *rRNA* sequences grouped with other *B. bigemina* reference sequences in a stable monophyletic cluster. Babesiosis caused by *B. bigemina* is frequently associated with cattle infection, causing fever, hemolytic anemia and even death. Together with *Babesia bovis* (not found in Portugal) and *B. divergens*, these *Babesia* species generate substantial economic and health losses in cattle production worldwide [44,45]. Although not recognized as a zoonotic agent, *B. bigemina* infection was recently reported in a 13-year-old patient with clinical signs of fever, chills, sweating, anorexia, general malaise, arthralgia, abdominal pain, myalgia and urinary incontinence with dark urine (a clinical picture of kidney failure) [46]. Such findings reinforce the importance of considering babesiosis an emerging/neglected risk to humans as well. Two other samples yielded expected detection for piroplasmids: one sample of *I. ricinus* (pool of five nymphs) for *Babesia* sp. (Figure 2) and one sample of *H. punctata* (female) for *Theileria* sp. (Figure 3). Such associations among these tick species and these TBPs have been previously reported [47,48,49,50]. Additionally, these *H. punctata* and *I. ricinus* specimens were collected at TNM and are in accordance with previous results regarding the epidemiology of both *Babesia* spp. and *Theileria* spp. within this region [36]. A significant note about *Theileria* spp., which are piroplasmids that belong to the apicomplexa phylum, is that these microorganisms are among the most important TBPs responsible for infecting cattle, leading to economic losses in European countries within the Mediterranean basin, but not responsible of causing any zoonosis [51]. It is noteworthy to mention that, at TNM, *B. bigemina* and *Babesia* sp. were detected infecting *I. ricinus* ticks, a very unusual relationship regarding the epidemiological literature, which can be explained due to the peculiar characteristics of this ecological area, as it has a rich and diverse fauna of vertebrate animals, including the fallow deer (*Dama dama*) and the red deer (*Cervus elaphus*), which are the most frequent wild hosts in nature for *I. ricinus* and probably influence the circulation and maintenance of these piroplasmids at this specific area [36]. Regarding the positive sample for *Babesia* sp. composed of a pool of five nymphs of *I. ricinus*, which did not reach a species level for the phylogenetic resolution, this sample was, afterward, submitted to a nested PCR assay as previously described [52], targeting a different fragment of the 18S *rRNA* gene. This analysis was not able to better characterize this pathogen, as the returned sequences showed a considerable number of low-quality base calls. For a better phylogenetic resolution, future and thorough studies with other markers should be carried out to clarify the result of this sample, since other samples in this study showed fine results at a species level resolution. An important finding in the present study pointed toward the infection of *R. pusillus* adult females, collected in Mata do Choupal, with an unknown piroplasmida microorganism (Figure 2) with identical similarity (100%) as some of the ones detected in previous studies [53,54,55]. The first Megablast hits of sequences that were obtained matched uncultured alveolates and uncultured eukaryotes from environmental samples. These studies have used generic and degenerate primers targeting different fragments of the 18S *rRNA* [53,54,55]. In the present study, using specific primers to detect piroplasmids, a single sequence was obtained that clustered with those of uncultured organisms, outside the *Babesia* monophyletic group, as displayed in the *Babesia* phylogenetic tree (Figure 2).

The prevalence of *N. mikurensis* in questing ticks reported here (14%) is within the range of previous studies that have shown a total infection rate of 0.1–24.2% regarding the analysis of questing ticks [21,56,57,58]. *Ixodes ricinus* is the main vector of *N. mikurensis* [56]. Besides this tick species, the present study also identified other tick species that, to the best of our knowledge, have not yet been reported to be infected by this bacterium (*D. marginatus*, *R. pusillus* and *R. sanguineus* s. l.). One of the possible explanations for these new discoveries could be that previous studies have primarily focused their efforts on collecting and screening *I. ricinus*, given the medical importance in Europe due to its anthropophilic behavior and its competence for the transmission of several TBP [30].

Targeting the Anaplasmatacea *16S* ribosomal gene provided additional findings to the present study with the detection of *M. mitochondrii*, an intracellular endosymbiont bacterium of hard and soft ticks [59]. First, it was reported to be associated with free-living *I. ricinus* females [60]. Since then, studies based on molecular evidence have reported anthropophilic and zoophilic ticks harboring *M. mitochondrii* such as *A. americanum*, *H. punctata*, *Ixodes holocyclus*, *Rhipicephalus bursa*, *R. sanguineus* s. l. and *Rhipicephalus turanicus* [61,62]. Other important aspects about *M. mitochondrii* include its vertical transmission capacity, as the bacterium reproduces mainly in the mitochondria of ovaries in *I. ricinus* females and is therefore maternally inherited [60]. Most recently, Guizzo et al. [63] studied the functional integration of *M. mitochondrii* into the biology of *I. ricinus*, and their conclusions indicate that *M. mitochondrii* represents an intrinsic component of tick ovarian tissue, and when absent, it results in the formation of substandard larvae with reduced capacity to blood-feed. This α-proteobacterium symbiont was detected in *I. ricinus* and *I. frontalis*, as previously reported [64,65,66,67]. Additionally, it was also detected in *D. marginatus*, in agreement with a previous study [68]. A recent report showed that this mitochondrial-residing bacterium affects oogenesis [69], but much remains to be known regarding the impact of its presence in ticks. Although this bacterium can be transmitted through tick bites [64], another study showed no clinical signs in people exclusively infected with *M. mitochondrii*; however, although it may not be harmful to humans, it could play a role in TBP transmission to mammalian hosts [62], since endosymbionts like *M. mitochondrii* often maintain a delicate microbial balance in ticks, hindering the presence of other microbes, blocking related pathogens or even enhancing their presence. A study on tick symbiont community structure showed a strong association between *Midichloria*, *Rickettsia* and *Coxiella* bacteria, but more profound studies are needed to clarify the functional role of this unique symbiont [70]. Additionally, the sequencing of amplicons derived from genetic material of three other *I. ricinus*, using the same primers, yielded unexpected results. Despite the small length of the obtained sequences (their submission to GenBank was, therefore, not possible), the level of coverage (approximately 66%), when compared with sequences previously deposited in GenBank, returned a hit with *Wolbachia* spp. (ID above 95%). These results can be explained by the primers utilized to detect Anaplasmataceae bacteria, which are generic [40]. In this sense, the present study suggests that *I. ricinus* can potentially harbor *Wolbachia* endosymbionts, but more studies need to be performed. Unfortunately, due to the lack of tick genetic material, further analyses were not possible. However, it is relevant to note that these results are ecologically supported by previous studies that have reported *I. ricinus* nymphs being parasitized by *Ixodiphagus hookeri* [71]. *Wolbachia* spp. are intracellular, Gram-negative, α-proteobacteria that are known to infect about 60% of arthropod species [72]. In insects, these endosymbionts directly reduce viral replication for dengue and West Nile viruses and act as a manipulator of host reproduction [72,73]. Although little is known about the effects that these bacteria may cause in ticks, they have already been reported to infect at least three tick species, namely: *I. ricinus*, *Rhipicephalus microplus* and *A. americanum* [74,75,76]. Furthermore, a previous study identified that both *Wolbachia* sequences (*wsp* and *ftsZ* genes) were identical to those already reported, such as that associated with an endoparasitoid wasp named *Ixodiphagus hookeri*. It is noteworthy to mention that this endoparasitoid is known to emerge from engorged nymphs of *I. ricinus*, indicating that this tick species is not the natural host of these endosymbionts [71]. Therefore, more studies on this subject would certainly help to increase information on how *Wolbachia* endosymbionts would act as a possible biological control, preventing the transmission of TBPs.

Most of the positive samples screened for TBPs that belong to the Anaplasmataceae family did not yield the expected phylogenetic resolution. Such results were not expected, as *Anaplasma* spp. and *Ehrlichia* spp. have been detected in ticks using the same primers and PCR protocol [40]. Regarding the molecular marker used for screening piroplasmids, similar outcomes were achieved, albeit at a significantly lower frequency. Alveolates and eukaryotic microorganisms were detected with a distinct molecular and phylogenetic resolution. Therefore, it is recommended to use more stringent molecular markers in future studies to attain a finer resolution regarding these findings.

## 5. Conclusions

The present study revealed the presence of *Babesia* spp., *Theileria* spp., *Neoehrlichia mikurensis* and *Midichloria mitochondrii* DNA in ticks from five different ecological areas in mainland Portugal. The findings, supported by previous reports, include the detection of *Babesia* sp. and *Theileria* sp. in *I. ricinus* and *H. punctata*, respectively; the infection of *I. ricinus* by *N. mikurensis*; and the detection of *M. mitochondrii* in both *I. ricinus* and in *I. frontalis*. To the best of our knowledge, this study reports for the first time the detection of *N. mikurensis* infecting *D. marginatus*, *R. pusillus* and *R. sanguineus* s. l. Furthermore, our data suggest the possible presence of *Wolbachia* endosymbionts in association with *I. ricinus*. This is primarily due to the ecological connection between this tick species with *Ixodiphagus hookeri*, a parasitoid wasp specialized in parasitizing larvae and nymphs of Ixodidae ticks. As a result of this relationship, it becomes feasible to detect *Wolbachia* endosymbionts in *I. ricinus*. Some of the most interesting outcomes included the detection of *B. bigemina* infecting *I. frontalis*, *I. ricinus* and *R. sanguineus* s. l. and the detection of *N. mikurensis* infecting *D. marginatus*, *R. pusillus* and *R. sanguineus* s. l. This molecular evidence suggests that these ticks could be capable of maintaining the circulation of these TBPs in an endemic area associated with vertebrate hosts that are often parasitized by these tick species; nevertheless, it is not known whether these ticks can transmit these TBPs vertically or horizontally. Thus, studies about tick transmission capacity could elucidate some of these issues. Regarding studies that address TBP screening, the present study suggests that specific molecular markers should be applied to achieve not only clearer detection results but also a better phylogenetic resolution. Furthermore, epidemiological surveillance studies on neglected TBPs should be carried out, especially when associated with the collection of anthropophilic ticks, as they are still one of the best sources of mapping and monitoring eco-epidemiological updates, aiding medical diagnoses, the creation of risk maps and the development of predictive risk models that are constantly changing due to several biotic and abiotic factors toward a One Health perspective.

## Figures and Tables

**Table 1:** Primer sets used for tick-borne pathogen screening.

Method	Target Gene	Primer/Probe	Sequence 5′-3′	Product Size (bp)	Reference
PCR	18S *rRNA*	PIRO-A	AATACCCAATCCTGACACAGGG	408	[39]
		PIRO-B	TTAAATACGAATGCCCCCAAC		
PCR	16S *rRNA*	EHR16SD	GGTACCYACAGAAGAAGTCC	345	[40]
		EHR16SR	TAGCACTCATCGTTTACAGC		
qPCR	*IS1111*	Cox-F	GTCTTAAGGTGGGCTGCGTG	295	[41]
		Cox-R	CCCCGAATCTCATTGATCAGC		
		Cox-TM	FAM-AGCGAACCATTGGTATCGGACGTTTATGG-TAMRA		
qPCR	16S *rRNA*	Neo_16S_F	GTAAAGGGCATGTAGGCGGTTTAA	107	[42]
		Neo_16S_R	TCCACTATCCTCTCTCGATCTCTAGTTTAA		

**Table 2:** Hard tick species and specimens screened for Piroplasmida microorganisms and Anaplasmataceae bacteria.

Collection Site	Tick Species (Number of Specimens/Stage *)	No. of Infected Samples/No. of Tested Samples (%)		
		**Piroplasmida**	***Anaplasma* spp./*Ehrlichia* spp.**	***N. mikurensis***
**Grândola**	*D. marginatus* (39A)	10/39 (25.6)	4/39 (10.3)	-
	*I. ricinus* (19A)	4/19 (21.1)	8/19 (42.1)	2/14 (14.3)
**Parque Nacional Gerês**	*D. marginatus* (2A)	0/2 (0)	0/2 (0)	0/2 (0)
**Serra do Bussaco**	*D. marginatus* (7A)	1/7 (14.3)	3/7 (42.9)	4/7 (57.1)
	*I. frontalis* (1A; 2N)	1/3 (33.3)	2/3 (66.7)	0/3 (0)
	*R. pusillus* (8A)	1/8 (12.5)	8/8 (100)	0/8 (0)
	*R. sanguineus* s. l. (15A)	1/15 (6.7)	3/15 (20)	4/15 (26.7)
**Mata do Choupal**	*D. marginatus* (1A)	1/1 (100)	1/1 (100)	0/1 (0)
	*R. pusillus* (17A)	5/17 (29.4)	6/17 (35.3)	3/17 (17.6)
	*R. sanguineus* s. l. (3A)	0/3 (0)	0/3 (0)	3/3 (100)
**Tapada Nacional de Mafra**	*Haemaphysalis* spp. (4N)	1/1 (100)	1/1 (100)	-
	*H. inermis* (31A)	3/31 (9.7)	0/31 (0)	0/4 (0)
	*H. punctata* (19A; 16N)	3/22 (13.6)	5/22 (22.7)	0/7 (0)
	*H. lusitanicum* (17A)	0/17 (0)	13/17 (76.5)	-
	*I. ricinus* (82A; 517N; 1L)	37/179 (20.67)	52/179 (29.05)	9/97 (9.3)
	*R. sanguineus* s. l. (1A)	0/1 (0)	0/1 (0)	-
**Total**	**802 (262A; 118M, 144F); 539N; 1L**	**68/365 (18.6)**	**106/365 (29.0)**	**25/178 (14)**

* A-adult(s); M-male(s); F-female(s); N-Nymphs(s); L-larvae. *D. marginatus* = *Dermacentor marginatus*; *H. inermis* = *Haemaphysalis inermis*; *H. punctata* = *Haemaphysalis punctata*; *H. lusitanicum* = *Hyalomma lusitanicum*; *I. frontalis* = *Ixodes frontalis*; *I. ricinus* = *Ixodes ricinus*; *R. pusillus* = *Rhipicephalus pusillus*; *R. sanguineus* s. l. = *Rhipicephalus sanguineus* sensu lato.

## Data Availability

The data presented in this study are contained within the article.
